# Portugal’s voluntary food reformulation agreement and the WHO reformulation targets

**DOI:** 10.7189/jogh.09.020315

**Published:** 2019-12

**Authors:** Francisco Goiana-da-Silva, David Cruz-e-Silva, Luke Allen, Alexandre Morais Nunes, Conceição Calhau, Ana Rito, Alexandra Bento, Marisa Miraldo, Ara Darzi

**Affiliations:** 1 Centre for Health Policy, Institute of Global Health Innovation, Imperial College London, London, United Kingdom; 2 Faculdade de Ciências da Saúde, Universidade da Beira Interior, Covilhã, Portugal; 3 Center for Innovation, Technology and Policy Research, IN+, Instituto Superior Técnico, Universidade de Lisboa, Lisbon, Portugal; 4 Nuffield Department of Primary Care Health Sciences, University of Oxford, United Kingdom; 5 Centre for Public Administration and Public Policies, Institute of Social and Political Sciences, University of Lisbon, Lisbon, Portugal; 6 Nutrition and Metabolism, NOVA Medical School, Faculty of Medical Sciences, NOVA University of Lisbon, Lisbon, Portugal; 7 Center for Health Technology Services Research (CINTESIS), Porto, Portugal; 8 INSA, National Institute of Health, Lisbon, Portugal; 9 Portuguese Order of Nutritionists, Porto, Portugal; 10 Department of Management & Centre for Health Economics & Policy Innovation (CHEPI), Imperial College Business School, London, United Kingdom; 11 Department of Surgery and Cancer, Faculty of Medicine, Imperial College London, London, United Kingdom

In response to stalling progress in NCD related premature mortality, the Portuguese government declared the ‘Promotion of Healthy Eating’ a national priority and convened a multisectoral task force with representatives from several ministries in order to tackle unhealthy diets [1]. With the inputs from civil society, non-governmental organisations and health authorities, this task force developed the Integrated Strategy for the Promotion of Healthy Eating (*Estratégia Integrada para a Promoção da Alimentação Saudável – “*EIPAS*”*). The strategy – endorsed by the Portuguese Ministers Council in December 2017 – included fiscal measures, co-regulation agreements with the food industry, enhanced cooperation with municipalities, and measures to change the food environments in public settings among many other initiatives [1].

In December 2016, the Portuguese Parliament approved a special consumption tax on sweetened beverages which has been associated with product reformulation and a fall in sales [2]. Inspired by the success of the tax on sweetened beverages, in December 2018, the government proposed a tax on salty processed foods; as salt is the leading dietary risk factor for NCDs in Portugal [3]. Even though the average consumption is 10.7g/d [3] far above of the WHO recommended threshold of <5g/d, the majority of the Portuguese Parliament Members voted against this proposal, recommending instead a co-regulation agreement with the food industry to achieve similar changes in consumption of salt [4]. After one year of negotiations, the Portuguese Ministry of Health (MoH) and the food industry representatives signed a broad ‘Food Industry Co-regulation Agreement’ on the 2^nd^ of May 2019.

The agreement covers the main food products high in salt, sugar, and *trans* fatty acids as well as the main dietary sources of these nutrients for the Portuguese population.

An analysis of population consumption patterns [5,6], conducted by the *Institute of Public Health at the University of Porto*, led to a consensus among the different agreement stakeholders that 11 food categories should be subject to reformulation (**Table 1**). All stakeholders agreed that the consumption targets to be achieved should be based on a baseline assessment of sales figures for food products representing at least 80% of the market share for each category.

**Table 1:** Sugar, salt and trans fatty acids reduction targets before and after negotiations

Food category	Targets for macronutrient reformulation	
**Targets suggested by the MoH (before negotiations)**	**Targets agreed between the MoH and the food sector (after negotiations)**
**Sugar:**		
Breakfast cereals	20%	10%
Cookies and biscuits	20%	No agreement reached
Chocolate milk	20%	10%
Yogurts	20%	10%
Soft drinks	20%	10%
Fruit juices	20%	7%
**Salt:**		
Crisps and other snacks	16%	12%
Cookies and biscuits	16%	No agreement reached
Breakfast cereals	16%	10%
Processed meats (ham)	16%	No agreement reached
Cheese	16%	No agreement reached
Ready-to-eat soups	16%	10%
Bread Toast	16%	No agreement reached
Bread	30%	30%
**Trans fatty acids:**		
Fat spreads	<2g of fat	<2g of fat
Cookies and biscuits	<2g of fat	<2g of fat
Pastries	<1g of fat	<1g of fat

*All reductions percentages are based on baseline levels from March 2018.

The initial reformulation targets proposed by the MoH (before negotiations with the food industry) were based on the WHO salt reduction targets [7] as well as on other countries’ preliminary food reformulation experiences [8,9]. The final co-regulation agreement framework utilised the Nielsen Consumer Panels information namely monthly sales for every food product, brand and category as well as their respective nutritional information validated by the Health authorities. These sources were chosen in order to optimise compliance, transparency and accountability.

A joint Ministry of Health-WHO modelling exercise [10] suggests that fully meeting the targets initially suggested by the MoH (before negotiations with the food industry) would prevent 798 premature deaths from non-communicable diseases per year [11]. It concludes, however, that even by adopting such ambitious reformulation targets, Portugal is unlikely to achieve the WHO target of reducing premature deaths attributable to noncommunicable diseases by one third by 2030. Despite such evidence, after one year of negotiations, the Food Industry representatives have been able to persuade the MoH not only to delay the agreement targets deadline from 2021 to 2022, but also to lower the initial reformulation targets (**Table 1**). Since the final agreement targets are actually much less ambitious than the preliminary ones, the health impact of the agreement will be considerably smaller.

The projection that voluntary industry agreement will prevent a relatively small number of deaths is supported by previous evidence suggesting that voluntary industry action can achieve health gains. Nevertheless, voluntary action will be insufficient on its own and must be complemented with other public health interventions in order to substantively improve population health outcomes [12]. In fact, the literature strongly suggests that mandatory approaches generate larger health gains than voluntary agreements [12,13]. Cobiac and colleagues [10] estimate that health gains from mandatory measures could be 20 times higher than voluntary interventions [11].

**Figure:**
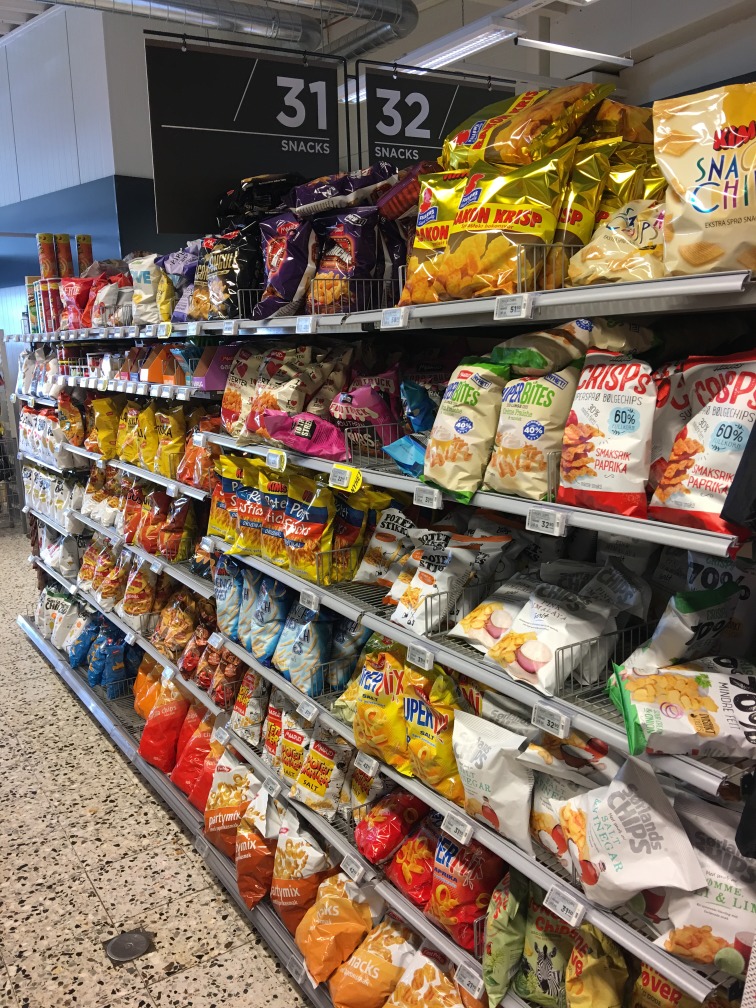
Photo: Wolfmann, CC BY-SA 4.0 (https://creativecommons.org/licenses/by-sa/4.0)

We are concerned that the consensus reformulation agreement targets achieved after negotiations with the food industry may not be ambitious and timely enough, neither to have an effective impact on the NCD epidemic, nor to allow Portugal to achieve the WHO food reformulation targets by 2030. Further research analysing the impact of the Portuguese Government’s flexibility during the negotiations with the processed food industry representatives would be important in order to promote accountability, to inform other policy makers facing similar negotiations and to conclude if such a limited agreement was a worth-while enterprise.

We have to acknowledge that engaging with the MoH and committing to voluntarily targets carries financial implications for industry in terms of reformulation costs, costs of engagement, and the risk of sales reductions as a result of any product changes. These factors should not obscure the fact that poor diet is a leading cause of death and disability, nor should weak voluntary efforts be allowed to trump effective legislative measures (including regulation and fiscal instruments) that are much more likely to improve diets and save lives. Given that the evidence suggests that even the stronger originally proposed voluntary targets would only prevent a small minority of diet-related deaths, we feel that the Government should immediately consider complimentary mandatory policies, including those that cover food served in cafeterias, canteens, restaurants and hotels as one of the leading sources of sugar, salt and trans fats in Portugal.
